# Gut-Homing Conventional Plasmablasts and CD27^−^ Plasmablasts Elicited after a Short Time of Exposure to an Oral Live-Attenuated *Shigella* Vaccine Candidate in Humans

**DOI:** 10.3389/fimmu.2014.00374

**Published:** 2014-08-20

**Authors:** Franklin R. Toapanta, Jakub K. Simon, Eileen M. Barry, Marcela F. Pasetti, Myron M. Levine, Karen L. Kotloff, Marcelo B. Sztein

**Affiliations:** ^1^Center for Vaccine Development, University of Maryland School of Medicine, Baltimore, MD, USA; ^2^Department of Medicine, University of Maryland School of Medicine, Baltimore, MD, USA; ^3^PaxVax, Inc.Redwood City, CA, USA; ^4^Department of Microbiology and Immunology, University of Maryland School of Medicine, Baltimore, MD, USA; ^5^Department of Pediatrics, University of Maryland School of Medicine, Baltimore, MD, USA

**Keywords:** *Shigella*, oral vaccine, plasmablasts, homing, B cells

## Abstract

Currently, there is no licensed *Shigella* vaccine; however, various promising live-attenuated vaccine candidates have emerged, including CVD1208S (Δ*guaBA*, Δset, Δ*sen S. flexneri* 2a), which was shown to be safe and immunogenic in Phase 1 clinical trials. Here, we report the immune responses elicited in an outpatient Phase 2 clinical trial in which subjects were vaccinated with CVD 1208S. Oral immunization with CVD 1208S elicited high anti-*S. flexneri* 2a LPS and IpaB antibody responses as well as an acute plasmablast (PB) infiltration in peripheral blood 7 days after immunization. PB sorted based on their expression of homing molecules confirmed that cells expressing integrin α4β7 alone or in combination with CD62L were responsible for antibody production (as measured by ELISpot). Furthermore, using high-color flow-cytometry, on day 7 after immunization, we observed the appearance of conventional PB (CPB, CD19^dim^ CD20^−^ CD27^+high^ CD38^+high^ CD3^−^), as well as a PB population that did not express CD27 (CD27^−^ PB; pre-plasmablasts). The pattern of individual or simultaneous expression of homing markers (integrin α4β7, CD62L, CXCR3, and CXCR4) suggested that CPB cells homed preferentially to the inflamed gut mucosa. In contrast, ~50% CD27^−^ PB cells appear to home to yet to be identified peripheral lymphoid organs or were in a transition state preceding integrin α4β7 upregulation. In sum, these observations demonstrate that strong immune responses, including distinct PB subsets with the potential to home to the gut and other secondary lymphoid organs, can be elicited after a short time of exposure to a *shigella* oral vaccine.

## Introduction

The intestine constitutes the largest immunological organ in the body and is home to the majority of lymphocytes (1, 2). In this organ, pathogens such as *Shigella* and *Salmonella* activate antigen presenting cells (APC) [e.g., dendritic cells (DC)] that migrate to mesenteric lymph nodes (MLN) where they stimulate lymphocytes (3). Subsequently, activated lymphocytes migrate to the intestinal lamina propria, as effector immune cells, largely via blood (2, 4–6). Lymphocytes homing to the intestine express both CCR9, a chemokine receptor mediating homing to the small intestine (1, 2, 4, 7, 8) and integrin α4β7 (9), a gut-specific homing receptor that recognizes Mad-CAM1 on the endothelial venules of the intestine. On the other hand, lymphocytes expressing CD62L (L-selectin) home preferentially to peripheral lymph nodes by binding to Gly-CAM1, which is found on high endothelial venules through which lymphocytes enter secondary lymphoid organs (10–12). Expression of these homing markers on lymphocytes is regulated by intestinal DC during antigen presentation (3–6).

Efforts are ongoing to better characterize the intestinally derived immune responses to *Shigella* infection. This information will be invaluable to guide the development of novel live oral vaccines against a pathogen whose global burden is well-documented (13, 14). After *Shigella* enters the host it infiltrates the gut epithelium, primarily via M cells (15–17). Innate immunity is often insufficient to clear a *Shigella* infection, particularly once it enters epithelial cells where this microorganism can move freely inside and between cells (18). As the infection progresses, *Shigella* activates the adaptive immune system and induces humoral (antibody) and effector T cell (CMI) responses, as well as memory (B and T) responses (15, 17–24). Various lines of evidence point to the importance of the B cell compartment in protection against repeated *Shigella* infections. For one, serotype-specific protection has been demonstrated in field (25–27) and clinical settings (28, 29) in humans and in primate studies (30). Serotype-specific IgA and IgG antibodies as well as circulating IgA antibody-secreting cells (ASC) directed against the (LPS) O-antigen have been correlated with protection (21, 23, 29–37). More recent evidence suggests that antibodies to invasion plasmid antigens (Ipa) also play an important role in protection (23, 24, 38–42). Among B cells, plasmablasts (PB) and plasma cells (PC) are responsible for antibody production/secretion. PB, which are not yet fully differentiated PC, proliferate extensively (43) and actively secrete antibodies while migrating to the target tissues where they will differentiate and home as PC. PB numbers peak in peripheral blood at day 6 or 7 following immunization or infection by *Shigella* (44) and other pathogens (43, 45–53). This transient peak of PB in peripheral blood, which coincides with the acute phase of immune responses, has been termed “acute PB infiltration” and used to differentiate from steady-state PB (54, 55). This “acute PB infiltration” has also been described in secondary infection/vaccination responses around the same time frame (7–9 days). While in a primary infection/vaccination, the “acute PB infiltration” is evidence of lymphocyte priming; in secondary infection/vaccination, it most likely indicates activation of memory B cells (B_M_). PB recently activated in the intestine, either by oral vaccination (19, 20, 56–59) or intestinal infections (60–62) are identified in circulation as ASC expressing integrin α4β7 (62, 63), implying a preferential homing of these cells to the intestinal lamina propria. Other homing markers, such as CXCR3 and CXCR4, which promote homing to inflamed tissues and bone marrow, respectively, have also been reported in PB cells (64).

Designing novel oral live-attenuated *Shigella* vaccine candidate strains requires striking an optimal balance between limited reactogenicity and induction of protective, long-lasting immunity. Due to the difficulty in achieving this fine balance, the goal of generating effective oral live-attenuated vaccine strains against *Shigella* has proven difficult, and occasionally resulted in vaccine-related reactogenicity. No licensed vaccines against *Shigella* are currently available (23, 24); however, various promising live-attenuated oral vaccine candidates have been developed. CVD 1208 and CVD 1208S, developed at the Center for Vaccine Development (CVD) of the University of Maryland, were constructed by introducing precise deletions in the wild-type *S. flexneri 2a* strain 2457T (65). Both vaccine strains are impaired in their ability to synthesize guanine nucleotides (Δ*guaBA*), and lack *Shigella* enterotoxins (ShET) 1 and 2, encoded by *set* and *sen*, respectively. After CVD 1208 showed promising results in clinical trials (66), it was reconstructed using animal-free media to comply with regulatory requirements for use in humans, and shown to be well-tolerated and immunogenic in Phase 1 clinical trials (65). Herein, we report the results of a Phase 2 clinical trial in which a new lot of CVD 1208S grown in an animal product-free medium different from the one used previously. After the first vaccine dose, some subjects experienced fever and/or diarrhea, which was attributed to the new soy-based medium used to grow CVD 1208S. This prompted the initiation of antibiotic treatment 1–4 days post-immunization. Here, we describe the humoral immune responses and the homing patterns of PB cells elicited in these volunteers, as well as a PB population that lacks CD27 expression (CD27^−^ PB) and appears to migrate to lymphoid organs different from “conventional” PB (CPB) cells.

## Materials and Methods

### Subjects and study design

Healthy male and non-pregnant female volunteers aged 18–49 years were recruited from the Baltimore/Washington, DC area for this randomized, placebo-controlled, and double-masked clinical trial (Table 1). Volunteers were required to be in good health as evidenced by medical history, physical examination, and laboratory evaluation, as previously described (65, 66). Serial cohorts of volunteers who satisfied eligibility criteria and provided informed, written consent were randomly allocated 5:1 to receive vaccine (*n* = 50) or placebo (*n* = 10). Each subject was scheduled to ingest a dose of vaccine or placebo on days 0, 28, and 56. An independent safety monitor was charged with reviewing safety data after each dose to recommend whether additional dosing was acceptable. This study was approved by the Institutional Review Board and registered on ClinicalTrials.gov (identifier NCT00866476). In this report, we include the results of two cohorts (20 volunteers) who received a single dose of CVD 1208S (*n* = 17) or placebo (*n* = 3).

**Table 1:** **Summary of the demograpics of the volunteers recruited for the study**.

	Vaccine	Placebo	Total
	(*n*^a^ = 17)	(*n* = 3)	(*n* = 20)
**Age^b^**	37.2	32.3	36.5
(95% CI^c^)	(33.2–41.1)	(12.1–52.6)	(32.8–40.1)
**Weight^d^**	182	222	188
(95% CI)	(165–200)	(24–421)	(168–208)
**BMI^e^**	28	32	28
(95% CI)	(25–31)	(1–62)	(25–32)
**Female^f^**	35	0	30
(95% CI)	(13–58)	(0–0)	(10–50)
**Black^f^**	82	67	80
(95% CI)	(64–100)	(13–100)	(62–98)
**Asian^f^**	0	33	5
(95% CI)	(0–0)	(0–87)	(0–15)
**White^f^**	18	0	15
(95% CI)	(0–36)	(0–0)	(0–31)

*^a^Number of volunteers*.

*^b^Mean in years*.

*^c^Confidence Interval*.

*^d^Mean in pounds*.

*^e^Body mass index*.

*^f^Percentage*.

#### Halting rules

Antibiotic treatment (ciprofloxacin 500 mg by mouth twice daily for 5 days) was not to be routinely administered to subjects unless specific reactogenicity events occurred and were considered to be vaccine-related: (1) one or more subjects experienced a serious adverse reaction, or (2) two or more subjects experienced any of the following adverse reactions: (a) fever >39°C; (b) passage of >5 diarrheal stools per day or requiring >2 L of intravenous hydration); or (c) passage of moderate dysentery (>2 dysenteric stools per day). If these events occurred, under advisement of the Safety Monitoring Committee, further doses of vaccine would not be administered.

### CVD1208S vaccine construction and preparation

CVD 1208S (Δ*guaBA*, Δ*sen*, Δ*set*) was constructed from wild-type *S. flexneri* 2a strain 2457T by a series of allelic exchange reactions using suicide plasmid deletion cassette technology as previously described (65). The vaccine was administered as a freshly harvested formulation. The inocula were derived from frozen master cell banks (MCB) prepared as previously described (65), except for the change in solid media from Hy-Soy (Kerry BioScience, Norwich, NY, USA) to APF Athena Lennox (Athena Enzyme Systems, Baltimore, MD, USA). In short, a frozen vial of master seed was plated onto APF LB agar plates (Lennox) containing Congo red dye (0.01%) (Sigma-Aldrich, St. Louis, MO, USA) and guanine (0.005%) (Sigma-Aldrich) (no antibiotics). After incubation (18–24 h, 37°C), single Congo red positive colonies were confirmed to be *Shigella* using antiserum (Denka Seiken, Niigata, Japan). Several colonies were suspended in sterile saline, inoculated into guanine-supplemented APF LB agar plates, incubated overnight (37°C) in the absence of antibiotics and harvested into sterile phosphate buffered saline (PBS, pH 7.4) (Sigma-Aldrich). The bacterial suspension was diluted (sterile PBS) to have an O.D. (660 nm) corresponding to the desired bacterial count per milliliter. The inocula were used within 4 h of preparation. The geometric mean of replicate colony counts performed before and after vaccination confirmed that the expected inoculum (1 × 10^9^ CFU per milliliter) of vaccine had been attained. Placebo consisted of buffer solution mixed with cornstarch USP (ScicenceLab.com Inc., Houston, TX, USA) added to match the turbidity of the vaccine (65).

Twenty outpatient volunteers were fasted 90 min before drinking 120 ml of a solution containing 2 g of NaHCO_3_ in 150 ml distilled water to buffer gastric acid. One minute later, the volunteers took the vaccine (at ~10^9^ cfu) in the remaining 30 ml of buffer or placebo (buffer alone). The volunteers were fasted an additional 90 min after vaccination. For the remainder of the day of vaccination and for the next 6 days, they recorded signs and symptoms onto a standardized diary form which were graded for severity, including their daily oral temperature, the occurrence of constitutional symptoms (headache, anorexia, malaise, and abdominal cramps), vomiting, and the character of all stools passed as either formed or loose (defined as stools, which take the shape of the container) and whether they contained visible blood. They returned to the study center on days 3 and 7 after vaccination to review diary cards. Antibiotics were given only in the event of unacceptable reactogenicity.

### Serum antibody responses

IgA and IgG antibody titers against LPS and recombinant *Shigella* IpaB were measured in serum before vaccination as well as on days 7, 14, and 28 post-immunization by enzyme-linked immunosorbent assay (ELISA) as previously described (65). Titers were calculated from linear regression curves and expressed as the reciprocal serum dilution that produced an O.D. of 0.2 above the blank, in ELISA Units (EU) per milliliter. Seroconversion was defined as a fourfold or higher rise in antigen-specific serum antibody post-vaccination compared to the pre-vaccination titer. The LPS from *S. flexneri* 2a LPS was purified by the hot aqueous phenol extraction method of Westphal (67) and the *Shigella* IpaB protein was purified as recombinant proteins expressed in *E. coli* as previously described (20, 21, 65, 68).

### Antibody-secreting cells

In order to evaluate intestinal priming by CVD 1208S, antigen-specific circulating IgA and IgG ASC were measured before (day 0) and 7 days after vaccination by enzyme-linked immunospot assay (ELISpot) as previously described (65, 66). In short, nitrocellulose plates (Millipore, Billerica, MA, USA) were coated with *Shigella* antigens including *S. flexneri* 2a LPS and *Shigella* IpaB (0.1 mg/ml), which were prepared according to previously published methods (20, 21, 65, 68). Coated plates were washed (3×) with PBS and blocked with 10% FBS RPMI (Gibco, Grand Island, NY, USA) for 2 h (5% CO_2_, 37°C). Peripheral blood mononuclear cells (PBMC) were then seeded (2.5 × 10^5^ per well) in quadruplicate and incubated for 14–16 h (5% CO_2_, 37°C). Subsequently, plates were washed with PBS-Tween (0.05%) and HRP-labeled goat anti-human IgA (1:2000) (Jackson ImmunoResearch Laboratories, West Grove, PA, USA) as well as IgG (1:1000) (Jackson ImmunoResearch Laboratories) were added to the respective plates and incubated for 1 h (37°C). Following a wash step, the 3-amino-9 ethylcarbazole C (AEC) substrate (Sigma-Aldrich) was added and plates incubated 10–20 min in the dark. Finally, plates were washed three times with distilled water to stop the reaction. Positive ELISpot responses were defined as a post-vaccination counts per 10^6^ PBMC that are at least three standard deviations (SD) above the mean pre-vaccination count for all subjects (in the log metric); a minimum of eight cells were required, which corresponds to the mean of medium negative control wells (two spots) plus three SD (66).

### Cell sorting strategy to evaluate expression of homing molecules by *Shigella*-specific ASC

Freshly isolated PBMC were stained with monoclonal antibodies to CD19-PE-Cy7 (clone J3–119, Beckman-Coulter – BC), CD27-PECy5 (Clone 1A4CD27, BC), CD62L-PE (Clone Dreg-56, Becton-Dickinson – BD), and integrin α4β7 (Clone ACT-1, Millenium: The Takenda Oncology Co., Cambridge, MA, USA) conjugated to Alexa 488 using an Alexa labeling kit (Molecular Probes). Cells were then simultaneously sorted into four populations: B naïve (Bn, CD19^+^ CD27^−^), (P) B memory (B_M_, CD19^+^CD27^+^) expressing CD62L but not integrin α4/β7 (CD62L^+^ α4β7^−^) (M) B_M_ expressing integrin α4β7 but not CD62L (CD62L^−^ α4β7^+^), or (M/P) B_M_ expressing both integrin α4β7 and CD62L (CD62L^+^ α4/β7^+^). Four-way sorting was performed in a MoFlo flow cytometer/cell sorter system (Beckman-Coulter). Purities of the sorted populations were 84.1–94%. The presence of *Shigella*-specific IgG and IgA ASC in each sorted population was measured as described above (see Antibody-Secreting Cells). The results are expressed as number of spots per 1 × 10^6^ cells.

### PB cell determinations by multichromatic flow cytometry

Peripheral blood mononuclear cells were isolated immediately after blood draws by density gradient centrifugation (69, 70). Freshly isolated PBMC were re-suspended in complete media [RMPI (Gibco, NY, USA) supplemented with 10% fetal bovine serum (FBS) (Gemini Bioproducts, West Sacramento, CA, USA), 2 mM l-glutamine (Gibco, Grand Island, NY, USA), 1× non-essential amino acids (Gibco, Grand Island, NY, USA), 10 mM HEPES (Gibco, Grand Island, NY, USA), 2.5 mM Sodium pyruvate (Lonza, Walkersville, MD, USA), 100 U/ml Penicillin, 100 μg/ml streptomycin (Sigma-Aldrich, St. Louis, MO, USA), 50 μg/ml Gentamicin (Gibco, Grand Island, NY, USA)] and stained for flow cytometry in 12 mm × 17 mm tubes using methods previously described (69, 71). Briefly, 1 × 10^6^ cells were plated and washed with flow cytometry staining (FC) buffer (4% FCS, 1× PBS) and 0.02 Sodium Azide). Cells were blocked with human IgG (50 μl of a 600 μg/ml solution in FC buffer) (Sigma, St Louis, MO, USA) and following one wash, stained with antibody cocktails prepared in FC buffer. Antibodies used included: IgD-FITC (Clone IA6-2,BD), CD38-PE (Clone HB7, BD), CD19-ECD (Clone J3-119; BC), CXCR3-PE-Cy5 (Clone 1c6/CXCR3, BD), CD20-PE-Cy7 (Clone L27, BD), CD3-Pacific Blue (Clone UCHT1, BD), CD14 (Clone TuK4, Caltag), integrin α4β7-Alexa 647 (Clone ACT-1); CD27-APC-Alexa700 (clone: 1A4CD27; BC), CD62L-APC-Alexa750 (Clone Dreg-56, Caltag), IgG-Biotin (Clone 12G5, BD), and IgA-Biotin (Clone G20-359, BD). Staining was performed at 4°C in the dark for 30 min. After washing the cells (2× with FC buffer), Pacific Orange–Streptavidin was added to each sample (Invitrogen, Carlsbad, CA, USA) (30 min) followed by two washes. Stained cells were fixed with 1% PFA in PBS and samples collected in a custom LSRII flow cytometer analyzer (BD, USA). Samples were analyzed using a FlowJo software package (Tree Star, USA).

### Statistical methods

For each randomized group, we calculated the proportion of subjects manifesting solicited clinical reactions and specific immunologic responses to vaccination. The geometric means and median values were calculated for serum antibody levels and ASC. Pre-vaccination and post-vaccination antibody titers and ASC counts at different time points were evaluated by one-way ANOVA with Dunnett’s post-test using day 0 as comparator. Post-vaccination ASC counts (D7) and serum antibody titers (D14) in each reactogenicity group as well as homing of sorted ASC were compared using the Mann–Whitney test. The percentages of CPB cells and CD27^−^ PB cells, pre-vaccination and post-vaccination were compared using the Mann–Whitney test. Spearman’s correlation coefficient was used to assess relationships between ASC (day 7) and the percentage of CPB cells and CD27^−^ PB cells (day 7). The same correlation test was used to assess the relationship between antibody titers and the percentage of CPB or CD27^−^ PB cells (day 7). Comparison of multiple homing markers expressed by CPB and CD27^−^ PB cells was done by one-way ANOVA with Bonferroni’s post-test. Microsoft Excel, GraphPad Prism, NCSS, and STATA were used for statistical analysis. All hypotheses were evaluated using two-sided tests. Two-sided *p*-values <0.05, without adjustment for multiple comparisons, were considered statistically significant.

## Results

### Clinical observations

Two cohorts were inoculated. Cohort 1 was composed of seven vaccinees (estimated inoculum 2.5 × 10^9^ cfu) and one placebo recipient. Cohort 2 was composed of 10 vaccinees (estimated inoculum 3.3 × 10^9^ cfu) and two placebo recipients. Cohorts were immunized 2 days apart. The occurrence of fever (17.6%), diarrhea (23.5%), and/or vomiting (5.8%) (Table 2) met criteria for antibiotic administration. Cohort 1 and 2 received antibiotics 4 and 1–2 days after immunization, respectively. No further vaccinations were administered. Even though the study was terminated early and no more cohorts vaccinated, safety follow-ups, stool cultures for fecal shedding of vaccine (unpublished data), and immunologic assays on days 7 and 28 were collected in the two cohorts that received the vaccine.

**Table 2:** **Reactogenicity and humoral responses in vaccinated volunteers and placebo controls**.

		ID	Reactogenicity			Humoral immure responses							
			Fever	Vomiting	Loose Stools	Anti-LPS IgA		Anti-LPS IgG		Anti-IpaB IgA		Anti-IpaB IgG	
					
			°C	#Episodes^b^	#Episodes	ASC^c^	Ab titer FI^d^	ASC	Ab titer FI	ASC	Ab titer FI	ASC	Ab titer FI
			(Day)^a^	(Day)	(Day)	(Day 7)	(Day 14)	(Day 7)	(Day 14)	(Day 7)	(Day 14)	(Day 7)	(Day 14)
		1				**120**	3.1	**15**	2.3	0	1.2	0	1.0
		2				**323**	**7.5**	6	2.5	0	1.1	0	**10.9**
	**Cohort 1**	3^f^	38.8 (1)		5/4 (1/2)	**98**	**4.7**	**41**	1.5	0	**155.3**	0	**17.9**
		4			4 (2)	**1114**	**103.6**	**1052**	**15.1**	**107**	**14.2**	0	**22.7**
		6				**556**	**39.9**	**12**	**8.1**	0	1.5	0	**11.3**
		7			3 (1)	**592**	**13.0**	**139**	**6.4**	0	1.3	0	2.0
	
**CVD1208S**		8				**208**	**13.5**	**44**	3.2	**28**	2.4	**11**	2.3
		9				**566**	**22.9**	**77**	**5.0**	4	1.5	0	1.6
		10				**50**	1.3	2	1.6	0	0.9	0	1.1
		11				**88**	**4.1**	**37**	2.1	**26**	2.0	**13**	1.1
	**Cohort 2**	14				**76**	2.1	**55**	1.7	**40**	**4.2**	0	**6.7**
		15				**298**	2.6	**109**	**5.9**	0	1.0	0	0.5
		16				**51**	2.0	2	0.8	**56**	2.8	0	2.2
		17	38.2 (1)			**130**	**4.3**	**32**	2.2	**248**	**46.2**	0	**138.7**
		18				**236**	**113.3**	**34**	**4.1**	0	1.0	0	**6.5**
		19	39.3 (1)	>5/5 (1/2)	8/>5 (1/2)	**98**	2.5	**54**	1.9	**400**	**41.0**	0	**198.3**
		20				**738**	**38.2**	**342**	**4.0**	**288**	**106.0**	0	**94.5**
	
**Placebo**		5				0	1.3	0	1.1	0	1.2	0	1.1
		12				0	1.1	0	0.9	0	1.0	0	1.0
		13				0	0.9	0	0.9	0	1.5	0	0.5

*^a^Day(s) on which the clinical sign was recorded*.

*^b^Episodes in 24 h*.

*^c^ASC per 1 × 10^6^ cells on day 7 post-immunization. Bolded results indicate >8 spots per 1 × 10^6^ cells*.

*^d^Antibody titer fold-increase (FI) at peak post-immunization. Bolded results indicate >fourfold-increase (seroconversion)*.

*^f^This volunteers reported fever of 39°C and six loose stools on the phone, but recorded 38.8°C and five stools in the diary*.

### Anti-*Shigella* humoral immune responses induced by CVD 1208S

**Figure 1:**
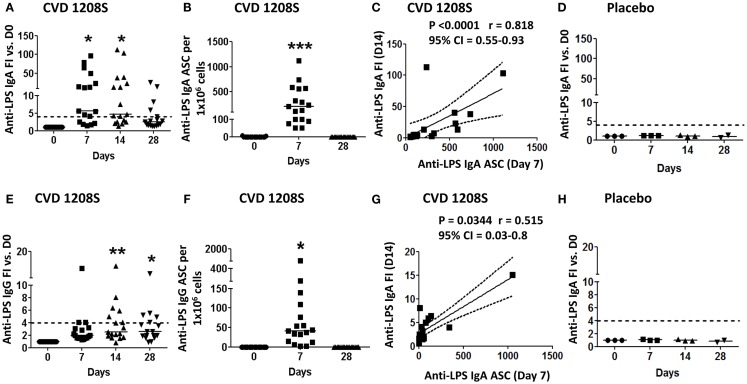
**Anti-LPS IgA and IgG immune responses to CVD 1208S**. **(A,E)** Anti-LPS IgA and IgG serum levels before and at days 7, 14, and 28 after immunization. Seroconversion was defined as *a* ≥ 4-fold-increase (dotted line) in antigen-specific antibody titers compared to day 0. **(B,F)** ASC anti-LPS IgA and IgG (ELISpot) before and at days 7 and 28 after immunization. **(C,G)** Spearman’s correlation between anti-LPS IgA and IgG antibody titers (fold-increase at day 14) and ASC (day 7). **(D,H)** Sera anti-LPS IgA and IgG in placebo volunteers (*n* = 3). In all plots each symbol indicates an individual volunteer. Dotted lines indicate seroconversion (fourfold-increase over day 0). Horizontal bars represent the median in all graphs. Asterisks show statistical significance when compared to day 0 (*p < 0.05; **p < 0.005; ***p < 0.0005).

CVD 1208S induced significant rises in anti-LPS IgA antibody titers compared to baseline at 7 and 14 days post-immunization, which began to diminish by day 28 (Figure 1; Table 2). By day 14 (peak day of anti-LPS IgA antibody production), 70.6% (12 of 17) of vaccinated volunteers had seroconverted (less than or equal to fourfold-increase (FI) over day 0) (Figure 1A). In contrast, placebo recipients did not develop anti-LPS IgA antibodies at any time point (Figure 1D). LPS-specific PB IgA ASC were detected 7 days post-immunization in all vaccinees (Figure 1B) but in none of the placebo recipients (data not shown). Moreover, LPS-specific IgA ASC was positively correlated with anti-LPS IgA antibody titers (at peak – D14) (Spearman’s correlation) (Figure 1C).

CVD 1208S also induced elevated anti-LPS IgG antibody titers; however, these were less robust than the IgA responses (Figure 1; Table 2). By day 14 (IgG peak), 41% of the vaccinated volunteers (7 of 17) had seroconverted (Figure 1E). IgG LPS-ASC were also identified on day 7 (Figure 1F) and the presence of these cells also correlated anti-LPS IgG (FI) at day 14 (Spearman’s correlation) (Figure 1G). Placebo recipients did not develop anti-LPS IgG responses at any time point (Figure 1H).

**Figure 2:**
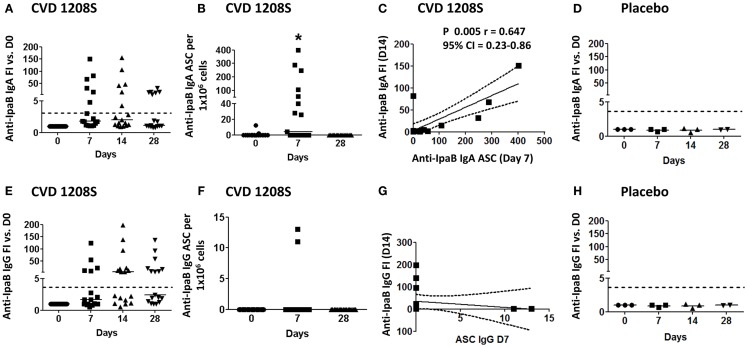
**Anti-IpaB immune responses to CVD 1208S**. **(A,E)** Anti-LPS IgA and IgG serological responses before and at days 7, 14, and 28 after immunization. **(B,F)** ASC anti-LPS IgA and IgG (ELISpot) responses, respectively, before and at days 7 and 28 after immunization. **(C,G)** Spearman’s correlation between anti-LPS IgA and IgG antibody titers (fold-increase at day 14) and ASC (day 7). **(D,H)** Sera anti-LPS IgA and IgG in placebo volunteers (*n* = 3). In all plots each symbol indicates an individual volunteer. Dotted lines indicate seroconversion (fourfold-increase over day 0). Horizontal bars represent the median in all graphs. Asterisks show statistical significance when compared to day 0 (*p < 0.05).

CVD 1208S also evoked antibodies (IgA and IgG) against the protein antigen IpaB; 35.3% (6 of 17) of the vaccinated volunteers seroconverted to IgA and 52.9% (9 of 17) to IgG by day 14 post-immunization (Figures 2A,E, respectively). Anti-IgA IpaB-ASC were also increased in peripheral blood (day 7) (Figure 2B) and the frequency of these cells correlated with the anti-IpaB antibody titers (day 7 FI over day 0) (Spearman’s correlation) (Figure 2C). On the other hand, anti-IgG IpaB-ASC were identified in only two volunteers (day 7) (Figure 2F) and no correlation with antibody titers was identified (Figure 2G).

The immune responses of volunteers that showed reactogenicity were compared to the vaccinees who remained asymptomatic. Although vaccinees without symptoms showed somewhat higher IgG and IgA seroconversions and ASC responses than vaccinees who exhibited reactogenicity, these differences did not reach a point of statistical significance (data not shown). Furthermore, to determine if the length of exposure to the vaccine had effects on the strength of the immune responses elicited, we compared cohorts 1 and 2. No significant differences in antibody titer or ASCs to LPS or IpaB were identified between these cohorts (data not shown).

### ASC expressing intestinal homing markers produce *Shigella*-specific antibodies

**Figure 3:**
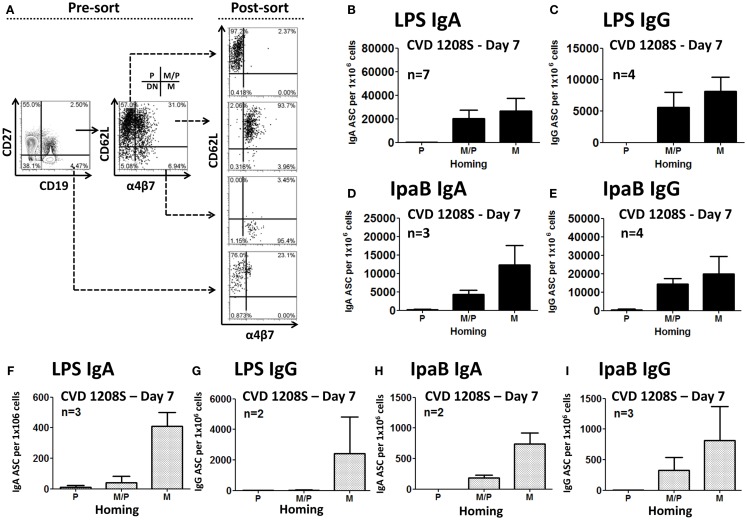
**Homing potential of sorted B cells**. **(A)** Freshly isolated PBMC (day 7) were stained with CD19, CD27, CD62L, and integrin α4β7 markers and then immediately sorted using a MoFlow cytometer (Beckman-Coulter) as depicted in the graph (for details see the Materials and Methods section). An aliquot of the sorted populations was run in the cytometer to confirm purity of the samples (>90%). As a negative control, naïve B cells (CD19^+^ CD27^−^), which had been demonstrated in previous experiments not to produce antibodies, were also sorted. P indicates cells expression of CD62L (homing to peripheral lymphoid tissues). M indicates expression of integrin α4β7 (homing to the gut). M/P indicates co-expression of integrin α4β7 and CD62L. DN cells, which did not express either integrin α4β7 or CD62L, were not sorted. Sorted cells were assayed for antibody production by ELISpot in plates coated with LPS or IpaB. Anti-LPS IgA **(B)** and IgG **(C)** ASC were identified in cells homing to gut mucosal tissues (M) and those with dual potential (M/P), but not in those expressing only CD62L (P). Similar results were shown in anti-IpaB IgA **(D)** and IgG **(E)**. **(F–I)** Results from similar sorting experiments using PBMC from a previous CVD 1208S Phase 1 vaccine trial. The numbers of volunteers (*n*) that responded to the stimulants and were used to generate each of the graphs are indicated.

To study the homing potential and antigen-specific production of PB cells (ASC), freshly isolated PBMC from day 7 post-immunization were fluorescently sorted into four populations of B cells (CD19^+^) based on the expression of CD27 and homing markers (integrin α4β7 or CD62L) (Figure 3A). B cells expressing CD27 and integrin α4β7, but not CD62L have the potential to migrate to the gut mucosa (M). On the other hand, B cells expressing CD27 and CD62L, but not integrin α4β7 migrate to peripheral lymphoid organs (P). Finally, B cells expressing CD27, integrin α4β7, and CD62L have dual migration potential (M/P). Sorted cells were then assayed for antibody production to specific *Shigella*-LPS by ELISpot. The results indicated that cells expressing integrin α4β7 alone (M) or in combination with CD62L (M/P), but not in those expressing only CD62L (P), were responsible for production of specific anti-*Shigella*-LPS IgA and IgG antibodies (Figures 3B–E). Control groups included naïve B cells (CD19^+^ CD27^−^) that did not produce antigen-specific antibodies (data not shown). Similar results, albeit of lower intensity, were observed in the prior Phase 1 clinical trial (CVD 24000) (65) in which subjects were immunized with CVD 1208S grown in Hy-Soy agar (Figures 3F–I).

### Increase in the frequency of conventional plasmablasts and CD27-plasmablasts following oral immunization with CVD 1208S

**Figure 4:**
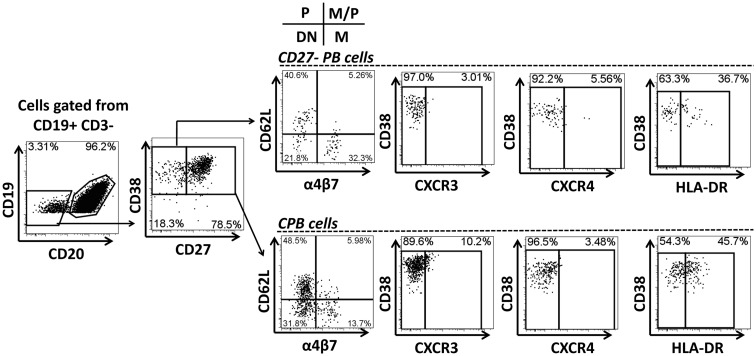
**Gating strategy for “conventional plasmablast” (CPB) and “CD27^−^ plasmablast” (CD27^−^ PB) cells**. Example of gating strategy used (day 0) in a representative volunteer. CPB cells were defined as CD19^dim^ CD20^−^ CD27^+(high)^ CD38^+(high)^ (IgD^−^ IgG^−^ IgA^−^). CD27^−^ PB cells were defined as CD19^dim^ CD20^−^ CD27^−^ CD38^+(high)^ (IgD^−^ IgG^−^ IgA^−^). Expression of the homing molecules integrin α4β7 and CD62L was determined in both subpopulations. P indicates cells expressing only CD62L, which home preferentially to peripheral lymphoid tissues. M indicates cells expressing only integrin α4β7, homing preferentially to the gut mucosa. M/P indicates cells co-expressing integrin α4β7 and CD62L (dual homing potential). DN indicates cells expressing neither CD62L nor integrin α4β7. Expression of other homing molecules including CXCR3 and CXCR4 as well as the activation marker HLA-DR were also evaluated in CD27-PB and CPB subsets.

**Figure 5:**
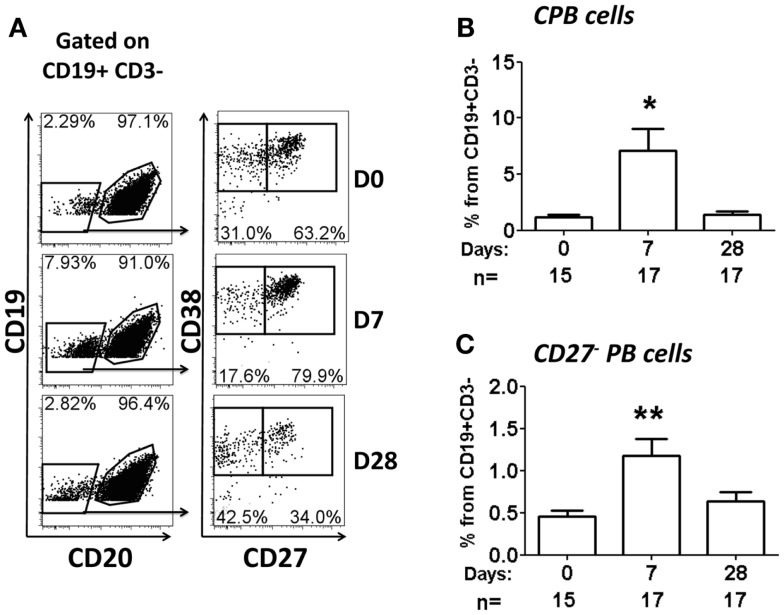
**Percentage of CPB and CD27^−^ PB cells following oral immunization**. **(A)** Example of population gating (CPB and CD27^−^ PB cells) at different time points (days 0, 7, and 28) in a representative vaccinated volunteer. **(B,C)** Show a compilation of the percentage of CPB and CD27^−^ PB (as percentage of CD19^+^ CD3^−^), respectively, from all vaccinees. Bars indicate arithmetic mean (±SEM). Asterisks show statistical significance when compared to day 0 (*p < 0.05; **p < 0.005).

Multichromatic flow cytometry was used to further analyze the expression of homing markers in PB cells. This technique allowed simultaneous evaluation of multiple homing markers, including integrin α4β7, CD62L, CXCR3, and CXCR4 as well as the activation marker HLA-DR. CPB cells were identified in freshly isolated PBMC and defined as CD19^dim^ CD20^−^ CD27^+(high)^ CD38^+(high)^ (Figure 4). These cells did not express surface Igs (IgD, IgG, and IgA, data not shown). Interestingly, another population of PB cells that lacked expression, CD27 (henceforth named CD27^−^ PB) was also observed. Similar to CPB cells, CD27^−^ PB cells did not express surface Igs (data not shown). Moreover, these cells were less frequent than CPB at day 0 (average fourfold lower frequency) (Figure 4 and data not shown). CPB and CD27^−^ PB cells showed a basal expression of HLA-DR as well as homing markers CD62L and integrin α4β7. Low basal expression of the homing markers CXCR3 and CXCR4 was also observed in both CPB and CD27^−^ PB cells (Figure 4). On day 7 post-vaccination, a statistically significant increase in the percentage of CPB and CD27^−^ PB cells (as percentage% of CD19^+^CD3^−^ cells), compared to day 0, was identified (Figure 5). However, CPB showed a higher increase than CD27^−^ PB cells (4-fold vs. 2.5-fold, respectively) (Figures 5A–C). By day 28, the percentage of CPB and CD27^−^ PB cells had returned to pre-vaccination levels (Figures 5B,C). CPB and CD27^−^ PB cells from placebo immunized volunteers did not show changes at any of the time points examined (data not shown).

Plasmablast cells have been reported to correlate with antibody production (Figures 2–4) (53, 72). However, because flow cytometry does not allow evaluation of antigen-specificity of these cells, we evaluated the relationship between the presence of PB cells and antigen-specific ASC by Spearman’s correlations. As expected, anti-LPS-ASC (IgA and IgG) correlated significantly with CPB cells (day 7, *P* < 0.05). Interestingly, the same correlation was found with CD27^−^ PBs (Table 3).

**Table 3:** **Correlation between anti-*Shigella*-LPS-ASC responses and changes in the percentages of CPB or CD27- PB cells**.

Correlated groups^a^	All volunteers		
	P^d^	r^e^	CI^f^
α-LPS IgA ASC (D7) and % CPB^b^ (D7)	0.0292	0.857	0.15–0.98
α-LPS IgG ASC (D7) and % CPB (D7)	0.0005	0.754	0.43–0.91
α-LPS IgA ASC (D7) and % CD27-PB^c^ (D7)	0.0312	0.5231	0.05–0.80
α-LPS IgG ASC (D7) and % CD27-PB (D7)	0.0103	0.604	0.17–0.84

*^a^Spearman’s correlation coefficient was used*.

*^b^% CPB = % of conventional plasmablast cells from the CD19^+^ gate*.

*^c^% CD27- PB = % of CD27- plasmablast cells from the CD19^+^ gate*.

*^d^*P* value*.

*^e^Spearman’s rho*.

*^f^Confidence Interval*.

### CVD 1208S increased expression of integrin α4β7, CXCR3, and HLA-DR in CPB and CD27-PB cells

**Figure 6:**
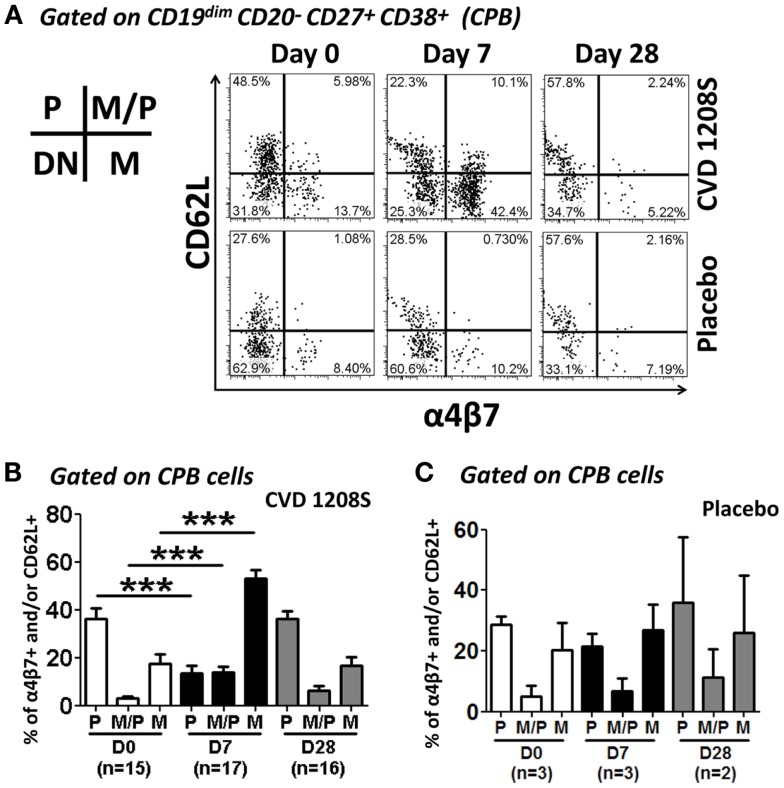
**Expression of homing molecules by CPB cells**. CPB cells [CD19^dim^ CD20^−^CD27^+(high)^ CD38^+(high)^] were gated as described in Figure 5 CPB cells were assayed for the expression of integrin α4β7 and CD62L to evaluate their homing potential. **(A)** Representative example of the changes in integrin α4β7 and CD62L expression in CPB cells at different time points (days 0, 7, and 28) in vaccine (top 3 panels) and placebo (bottom three panels). **(B,C)** Compiled data of the expression of homing receptors in CPB cells in vaccine and placebo recipients, respectively. Bars indicate arithmetic mean (±SEM). White, black and gray bars indicate days 0, 7, and 28, respectively. Horizontal lines indicate groups compared and asterisks indicate statistical significance (***p < 0.0005). In the inset: P indicates cells expression of CD62L only. M indicates expression of integrin α4β7 only. M/P indicates co-expression of integrin α4β7 and CD62L.

**Figure 7:**
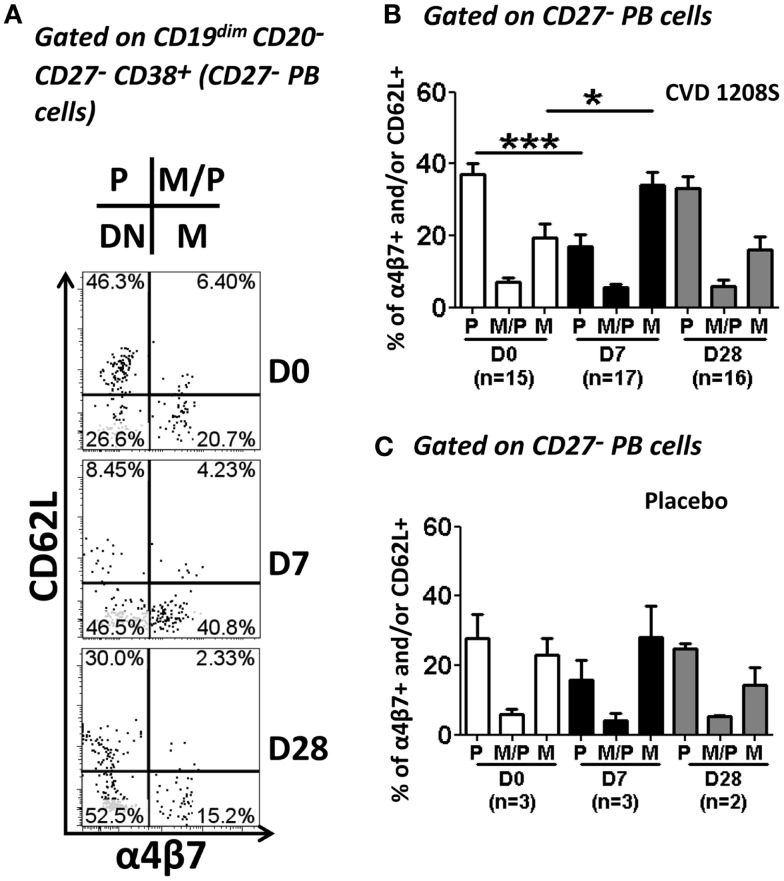
**Expression of homing molecules by CD27^−^ PB cells**. CD27^−^ PB cells [CD19^dim^ CD20^−^ D27^−^ CD38^+(high)^] were gated as indicated in Figure 4. Similar to CPB cells, CD27^−^ PB cells were evaluated for the expression of CD62L and integrin α4β7 to determine their homing potential **(A–C)**. **(A)** Representative example of the increase in integrin α4β7 and reduction of CD62L at day 7. **(B,C)** Compiled data of all vaccinated **(B)** and placebo **(C)** volunteers at each time point evaluated. White, black and gray bars indicate days 0, 7, and 28, respectively. Bars in plots indicate arithmetic mean (±SEM). Horizontal lines indicate groups compared and asterisk indicate statistical significance (*p < 0.05; ***p < 0.0005).

**Figure 8:**
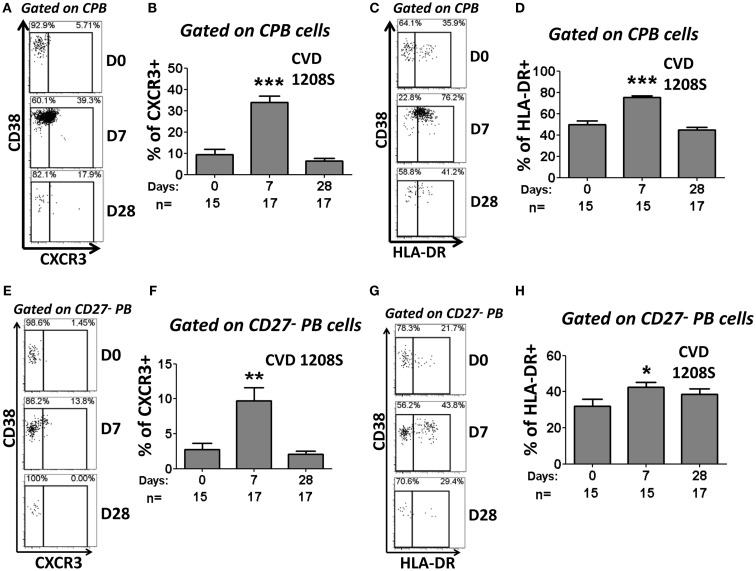
**Expression of CXCR3 and HLA-DR in CPB and CD27^−^ PB cells**. CPB and CD27^−^ PB cells (Figure 5) were also evaluated for the expression of CXCR3 and HLA-DR. Representative examples of the changes in CXCR3 **(A,E)** and HLA-DR **(C,G)** expression at the evaluated time points (Days 0, 7, and 28) are shown. Compiled data on the expression of CXCR3 **(B,F)** and HLA-DR **(D,H)** in CPB and CD27-PB are shown. Bars indicate arithmetic mean (±SEM). Asterisks indicate statistical significance when compared to day 0 (*p < 0.05; **p < 0.005; ***p < 0.0005).

**Figure 9:**
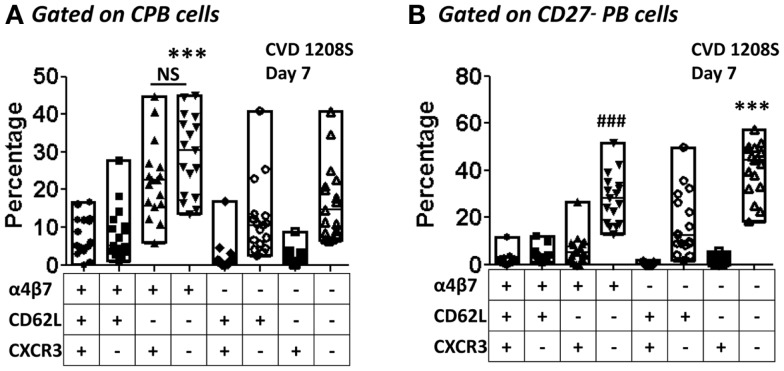
**Co-expression of homing molecules in CPB and CD27^−^ PB cells**. The expression of integrin α4β7, CD62L, and CXCR3 alone or in combinations (Boolean gates – FlowJo) were assayed in samples from day 7 in CPB and CD27^−^ PB gated cells [**(A,B)**, respectively]. The graph displays percentages of cells expressing each combination evaluated. Each symbol indicates an individual volunteer and in each group the bars indicate the highest and lowest percentages. Horizontal lines indicate the median. The groups were evaluated by one-way ANOVA, followed by Dunnet’s post-test using expression of integrin α4β7 alone as the control group. **(A)** ***p < 0.0005 integrin α4β7 alone versus all other groups except for integrin α4β7^+^CXCR3^+^. NS; not significant. **(B)** ^###^*p* < 0.0005 integrin α4β7 alone versus all other groups, except integrin α4β7^−^CD62L^−^CXCR3^−^, ***p < 0.0005 integrin α4β7^−^CD62L^−^CXCR3^−^ versus all other groups except the integrin α4β7^+^ alone.

Expression of CD62L and integrin α4β7 was evaluated in CPB and CD27^−^ PB cells (Figures 6 and 7, respectively). Using these markers, four different populations (P, M/P, M, and DN), each one with a different homing (migration) potential, were identified (Figure 6). On day 7 post-immunization, CPB and CD27^−^ PB from vaccinated volunteers showed a significant increase in the expression of integrin α4β7 (M) and down-regulation of CD62L (P) (Figures 6A,B). The percentage of cells with dual migration potential (M/P) increased only in CPB (Figures 6B and 7B). By day 28, expression of integrin α4β7 and CD62L had returned to their basal levels (Figures 6A,B and 7A,B). In contrast, these markers remained unaltered in placebo recipients at every time point assayed (Figures 6A,C and 7C). Expression of the chemokine receptor CXCR3 and HLA-DR were also significantly elevated in CPB and CD27^−^ PB cells on day 7 post-immunization and returned to pre-vaccination levels by day 28 (Figure 8). Expression of CXCR4 remained unchanged throughout the study (data not shown). Additionally, expression of integrin α4β7, CD62L, and CXCR3 alone or in combinations was assayed in CPB and CD27^−^ PB cells. This analysis was performed only on day 7 because this is the time point at which these markers showed significant changes. Expression of CXCR4 was excluded from the analysis since this marker did not show significant changes at the time points measured. The results indicated that CPB cells preferentially up-regulated integrin α4β7 alone and expression of this marker alone was statistically significant (*p* < 0.0005) when compared to all the other possible combinations, except for cells co-expressing integrin α4β7/CXCR3 (Figure 9A). CD27^−^ PB cells also preferentially up-regulated integrin α4β7 alone and this group was statistically significant compared to all other groups (except for α4β7^−^CD62L^−^CXCR3^−^) (Figure 9B). However, this group (integrin α4β7 alone) did not represent the most abundant cells since CD27^−^ PB that did not express any of the assayed homing markers, was the most abundant and statistically significantly different (*p* < 0.0005) from all other groups (including integrin α4β7 alone).

### Effect of reactogenicity and length of exposure to CVD 1208S on CPB and CD27^−^ PB cells

To determine if reactogenicity was associated with the frequency of CPB and CD27^−^ PB cells, we compared the percentage increases of these cells (day 7 post-immunization) between vaccinees who developed reactogenicity and those who did not. The results indicated that there were no statistically significant differences between these two groups (data not shown). Similar comparisons were performed for the other markers evaluated (α4β7, CXCR3, and HLA-DR) but no significant differences were identified. Finally, these markers in PB and CD27^−^ PB cells were also compared between volunteers of the first and second cohort and no statistically significant differences were identified (data not shown).

## Discussion

Two inpatient Phase 1 trials in which 14 healthy adults received ca. 10^9^ cfu of the live, attenuated freshly harvested *S. flexneri* 2a vaccine candidate CVD 1208 (grown in animal-containing media) (66) or CVD 1208S (grown in animal-free media) (65) suggested that deletions in *guaBA, sen*, and *set* resulted in a strain that was well-tolerated while retaining the ability to evoke an immune response. In the current study in which subjects received a freshly harvested vaccine formulation grown in an animal product-free medium not previously used in our clinical trials, a proportion of subjects experienced reactogenicity that was attributed the new growth medium. In fact, the reactogenicity reported in this manuscript was unexpected, since neither the previous two trials cited above (65, 66) nor a follow up clinical trial with CVD 1208S prepared under GMP (manuscript in preparation) resulted in any adverse effects. Even though the present trial was halted, it provided an unprecendented opportunity to study in depth the induction of humoral immune responses to *Shigella* after a brief (1–4 days) exposure to an attenuated vaccine before antibiotic treatment was initiated. Furthermore, this study allowed us to evaluate the homing patterns of PB (CPB and CD27^−^ PB cells) elicited by immunization.

Despite the short exposure time frame (4 days for cohort one and 1–2 days for cohort 2), CVD 1208S elicited both anti-LPS and anti-IpaB antibodies (IgA and IgG) with the LPS responses being more prominent (Figures 1 and 2; Table 2) (65). The cells responsible for the initial production of antigen-specific antibodies are PB (60) and consistent with this phenomenon, anti-LPS-ASC IgA and IgG (at day 7) correlated with anti-LPS IgA and IgG antibody titers (FI at day 14), respectively (Figures 1C,G). Similarly, anti-IpaB IgA ASC correlated with antibody titers (IgA at day 7) (Figure 2C). Anti-IpaB IgG ASC were detected only in a few volunteers, although significantly higher antibody titers were detected by ELISA. This suggests that there are still limitations in the assays used to detect PB, particularly for poorly immunogenic antigens (e.g., IpaB), and that a complete assessment of the immune responses can only be achieved by combining the results from multiple assays. In the present studies, anti-LPS responses appeared to be transitory since IgA and IgG titers increased on days 7 and 14, but began to decrease by day 28. On the other hand, anti-IpaB IgA and IgG antibody titers remained elevated up to day 28 (the last time evaluated). These results confirm and extend previously published data (65, 66).

The seroconversion rate induced by CVD 1208S in this trial (~70% for anti-LPS IgA) is similar to the one we previously reported for CVD 1208 (71%) (66) and higher than in a previous CVD 1208S trial (14%) (65). Therefore, these results indicate that in the present studies CVD 1208S appeared to be similar, or more, immunogenic than previous trials, suggesting that the enhanced immunogenicity could be attributed to reactogenicity; however, this does not appear to be the case since no differences in the strength of the immune responses were observed between the volunteers with and without reactogenicity. Additionally, CVD 1208S appears to be at least as immunogenic as other live-attenuated oral vaccines, such as Ty21a, the licensed oral attenuated typhoid vaccine, which typically exhibits a seroconversion rate of 40–60% (73–75).

Several reports have shown that PB cells appear in peripheral blood on day 7 post-infection or vaccination (45, 53, 60, 72). Consistent with these observations, we identified LPS- and IpaB-specific (IgA and IgG) ASC (ELISpot) on day 7 post-vaccination. Furthermore, PB cells are believed to ultimately migrate and home in the gut mucosa, the environment where they will produce antibodies. It is widely accepted that expression of integrin α4β7 promotes migration of cells to intestinal sites, while expression of CD62L promotes migration to peripheral lymphoid tissues, particularly lymph nodes. Consistent with this hypothesis, we found that sorted ASC that expressed either integrin α4β7 alone or in combination with CD62L, but not the ones that expressed only CD62L were responsible for antigen-specific antibody production, providing the first direct evidence of the relevance of gut-homing in subjects orally immunized with attenuated *Shigella* vaccines (Figures 3B–E). Of importance, similar results, albeit at lower intensity, were obtained in a previous Phase I clinical trial (CVD 24000) (Figures 3F–I).

We used multichromatic flow cytometry to characterize in depth the expression of homing markers by PB cells. Homing markers were assayed at various time points (days 0, 7, and 28) to determine differences in expression as the humoral immune responses were developing. PB cell were defined as CD19^dim^ CD20^−^ CD27^+(high)^ CD38^+(high)^ CD3^−^ and referred to as CPB, due to the identification of a second group of PB cells that lacked the expression of CD27, which were referred as CD27^−^ PB (Figure 4). CPB cells not only increased in frequency by day 7 post-immunization (Figures 5A,B), but also exhibited increased expression of integrin α4β7, while the expression of CD62L declined, further supporting the hypothesis that these cells ultimately migrate to the intestinal mucosa (Figures 6A,C). Another homing marker that was increased in CPB cells on day 7 was CXCR3, which promotes migration to inflamed tissues (Figures 8A,B) (50, 64). Thus, it is reasonable to speculate that following vaccination with an attenuated strain of *Shigella*, a mild inflammation of the gut mucosa would further promote CPB cells to home in those sites. Not surprisingly, CPB cells also showed increased levels of activation on day 7 post-vaccination as evidenced by the upregulation of HLA-DR (Figures 8C,D), a phenomenon also associated with differentiation into PC (54). Interestingly, no changes were noted in the expression of CXCR4 (data not shown), a chemokine that favors homing in the bone marrow. This observation suggests that few, if any, of the CPB induced by a mucosal pathogen (or vaccine) will home in the bone marrow. The preferred homing site of these cells remains to be identified. The lack of expression of CXCR4 and increased expression of integrin α4β7 also suggest that most CPB produce IgA as proposed by recent studies (54). Spearman’s correlation analysis suggested that the CPB cells identified by flow cytometry were indeed those detected by ELISpot assays (ASC) (Table 3). Finally, analysis of co-expression of homing molecules indicated that the vast majority of these cells express either integrin α4β7 alone or in combination with other homing marker(s) (Figure 9), further confirming that these cells are likely to migrate to the intestinal mucosa and inflamed tissues (combined CPB expressing integrin α4β7 alone or with other markers was found to be ~80). Therefore, it appears that only ~20% of CPB elicited after mucosal immunization migrate to tissues other than the intestinal mucosa.

Interestingly, we identified a population of PB that lacked CD27 expression (CD27^−^ PB; CD19^dim^ CD20^−^ CD27^−^ CD38^+(high)^; Figure 4). These cells are likely to represent the recently described pre-plasmablast population (76, 77). However, to our knowledge, this is the first report describing that these cells are elicited by oral immunizations in humans. CD27^−^ PB cells were less frequent than CPB (Figure 5C) and showed a somewhat similar pattern of expression of homing and activation markers than CPB (Figures 7–9). Despite the fact that integrin α4β7 was also an important homing marker (alone or combined ~30–35%) in CD27^−^ PB cells, in contrast to CPB, the largest percentage of CD27^−^ PB expressed none of the homing markers examined (~40–50% were quadruple negative for integrin α4β7, CD62L, CXCR3, and CXCR4) (Figure 9). This suggests that even though some CD27^−^ PB cells home in the intestinal mucosa, about half of these cells home in a different lymphoid organ (likely not the bone marrow), which at present remains unknown. Alternatively, since CD27^−^ PB are a pre-plasmablast population, the lack of expression of integrin α4β7 might be a consequence of their immature state and when these cells differentiate into CPB, increased expression of integrin α4β7 facilitates migration to the gut.

The severity of the disease induced by natural *Shigella* infections could have an impact on the strength of the immune responses elicited; however, it is unknown whether more severe disease (or vaccine reactogenicity) is associated with either enhanced or reduced immunogenicity. This is particularly important in vaccine development, since the goal is to develop immunogenic vaccines that do not produce reactogenicity; however, this balance has been difficult to achieve for *Shigella*. Since some vaccinated volunteers exhibited side effects, we investigated whether there was an association between reactogenicity and the strength and quality of the humoral immune responses elicited. Interestingly, no statistically significant differences were observed among the subjects who developed symptomatology and those who remained asymptomatic for any of the parameters evaluated (e.g., antibody titers, ASC frequencies, homing potential of ASC, degrees of activation), although the sample sizes were small. Another interesting observation derived from these studies relates to the early antibiotic treatment, which was initiated 1–4 days after immunization in all volunteers. Based on the strong humoral immune responses observed, it is reasonable to hypothesize that a relatively short period of exposure to an attenuated *Shigella* vaccine (~1–4 days) is sufficient to elicit strong immunity. A recent report in the *S. typhimurium* mouse model suggested that oral treatment with ciprofloxacin resulted in impaired development of the adaptive immunity (78). However, this does not appear to be the case in the current study since oral antibiotic treatment with ciprofloxacin (500 mg BID for 5 days) did not impair the development of the strong humoral immune responses.

In sum, we have shown the induction of robust antibody and ASC responses in subjects orally immunized with the attenuated *Shigella* vaccine strain CVD 1208S and characterized in detail the kinetics and homing potential of CPB and CD27^−^ PB elicited by vaccination. The observations that strong immune responses were elicited in individuals who did not show reactogenicity and that a short period of exposure (1–4 days) was sufficient to elicit appropriate humoral immunity, provides further impetus for the continuing development of attenuated *Shigella* strains to be used as oral vaccines.

## Conflict of Interest Statement

The Review Editor David J. M. Lewis declares that, despite having collaborated with author Myron M. Levine, the review process was handled objectively and no conflict of interest exists. Dr. Myron M. Levine, a co-author in this manuscript, is co-inventor in a patent for the development of Gua mutants of *Shigella* spp. licensed to PATH. The other co-authors declared no conflict of interest.
